# Clinical application of the paraspinal erector approach for spinal canal decompression in upper lumber burst fractures

**DOI:** 10.1186/s13018-014-0105-4

**Published:** 2014-11-13

**Authors:** Xi-Yan Xu, Zheng-Jian Yan, Qing Ma, Liang Chen, Zhen-Yong Ke, Fu Chen, Yun Chen, Lei Chu, Zhong-Liang Deng

**Affiliations:** Department of Orthopaedic Surgery, The Second Affiliated Hospital, Chongqing Medical University, 76 Linjiang Road, Chongqing, 400010 China; Department of Pharmacy Practice, University at Buffalo, Buffalo, NY USA

**Keywords:** Erector spinae, Direct spinal canal decompression, Minimally invasive spine surgery, Surgical approach, Upper lumber burst fractures

## Abstract

**Objective:**

Percutaneous pedicle screw fixation is commonly used for upper lumber burst fractures. The direct decompression remains challenging with this minimally invasive surgery. The objective was to evaluate a novel paraspinal erector approach for effective and direct decompression in patients with canal compromise and neurologic deficit.

**Method:**

Patients (*n* = 21) with neurological deficiency and Denis B type upper lumbar burst fracture were enrolled in the study, including 14 cases in the L1 and 7 cases in the L2. The patients underwent removal of bone fragments from the spinal canal through intervertebral foramen followed by short-segment fixation. Evaluations included surgery-related, such as duration of surgery and blood loss, and 12-month follow-up, such as the kyphotic angle, the height ratio of the anterior edge of the vertebra, the ratio of sagittal canal compromise, visual analog scale (VAS), Oswestry Disability Index (ODI), and Frankel scores.

**Results:**

All patients achieved direct spinal canal decompression using the paraspinal erector approach followed by percutaneous pedicle screw fixation. The mean operation time (SD) was 173 (23) min, and the mean (SD) blood loss was 301 (104) ml. Significant improvement was noted in the kyphotic angle, 26.2 ± 8.7 prior to operation versus 9.1 ± 4.7 at 12 months after operation (*p* <0.05); the height ratio of the anterior edge of the injured vertebra, 60 ± 16% versus 84 ± 9% (*p* <0.05); and the ratio of sagittal canal compromise, 46.5 ± 11.4% versus 4.3 ± 3.6% (*p* <0.05). Significant improvements in VAS (7.3 ± 1.2 vs. 1.9 ± 0.7, *p* <0.05), ODI (86.7 ± 5.8 vs. 16.7 ± 5.1, *p* <0.05), and Frankel scores were also noted.

**Conclusions:**

The paraspinal erector approach was effective for direct spinal canal decompression with minimal injury in the paraspinal muscles or spine. Significant improvements in spinal function and prognostics were achieved after the percutaneous pedicle screw fixation.

## Introduction

Approximately 10%–20% of spinal fractures are burst fractures mainly in the T11-L2 [1,2]. The three-column spine concept [3] indicated that burst fracture might result from failure under axial load of both the anterior and the middle columns, which lead to a narrowed spinal canal and injured nerve roots and a variety of neurological deficiencies. It is critical for patients with burst fracture to undergo spinal canal decompression in order to improve thoracolumbar spinal function and stability [4].

Indirect and direct spinal decompressions are commonly used in practice. Although remarkably different in procedures, the main purpose of these decompression approaches is intended to limit secondary injury to the spinal cord and improve neurological recovery after acute spinal cord trauma [5]. The traditional indirect decompression includes disc/ligaments stretch reduction, laminectomy, and partial removal of the pedicles [6,7]. In some patients, indirect approaches might not be sufficient and therefore, direct approaches are needed for complete decompression of the spinal canal. Since lateral operation for further decompression may substantially increase the risk of direct spinal injury at L2 and above, it is generally not recommended in neurosurgical practice [6].

The most common direct spinal canal decompression is accomplished through an anterior approach. The objective of this study was to investigate the effectiveness of a new dorsal lateral approach in terms of direct decompression. The neurological outcomes after the lateral paraspinal erector approach followed by minimally invasive posterior percutaneous pedicle screw fixation were also evaluated in patients with Denis B type burst lumbar fractures.

## Patients and methods

### Patients

The study recruited subjects with the following inclusion criteria: (1) age 18–80 years, (2) Denis B type burst fracture of the thoracolumbar spine (L1 or L2), (3) presence of neural deficits, and (4) >30% compromise of the spinal canal by vertebral fragments. The subjects with one of the following conditions were excluded: (1) no observed neural deficits, (2) spinal trauma or surgery history, (3) multiple-level burst fractures, and (4) coagulation disorders. The study was approved by the Institutional Review Board and the Ethics Committee at the Second Affiliated Hospital of Chongqing Medical University, and written informed consent was obtained from each subject.

A total of 21 subjects were enrolled in the study (13 males and 8 females) with an average age of 55 years (range 38–74 years). The presentation to the clinic was within 3–8 days of injury. Causes of injury included fall (*n* = 14) and car accident (*n* = 7). The Denis B type burst fractures (L1 = 14 cases and L2 = 7 cases) were diagnosed and confirmed using X-ray, CT scan, and magnetic resonance imaging (MRI).

### Surgical procedures

**Figure 1:**
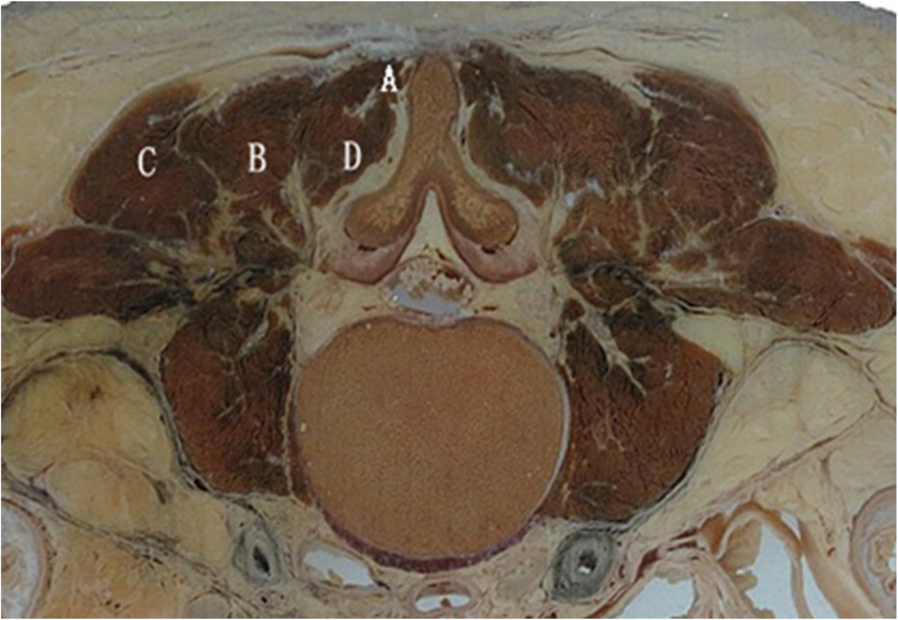
**The schematic diagram of upper lumbar erector spinae.** A: Spinalis. B: Longissimus. C: Iliocostalis. D: Multifidi.

**Figure 2:**
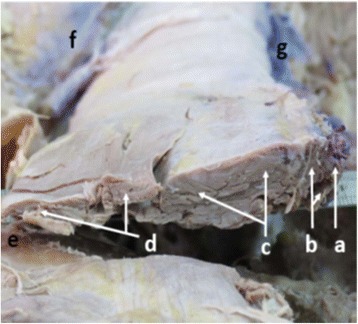
**The cross section of the erector spinae.** a: Spinalis. b: Multifidi. c: Longissimus. d: Iliocostalis. e: Costal bone. f: Posterior iliac crest. g: Spinous process.

All procedures were carried out under controlled general anesthesia with endotracheal intubation in the prone position on a radiolucent operating table. Paraspinal surgery was performed using the S4® spinal system (Aesculap Implant Systems, Center Valley, PA, USA). Pedicle screws were implanted into the adjacent vertebrae above and below the fracture. Unilateral or bilateral pedicle screws were implanted into the fractured vertebra based on the integrity of the pedicle. Direct spinal canal decompression was performed using a dorsal lateral approach next to the erector spinae; bony fragments in the spinal canal were removed. Our previous studies provided basic anatomic information in Chinese for the development of this approach [8]. The erector spinae at the upper lumbar was composed of the spinalis, longissimus, and iliocostalis (Figures 1 and 2). The starting point of this paraspinal erector approach is the lateral edge of the iliocostalis.

**Figure 3:**
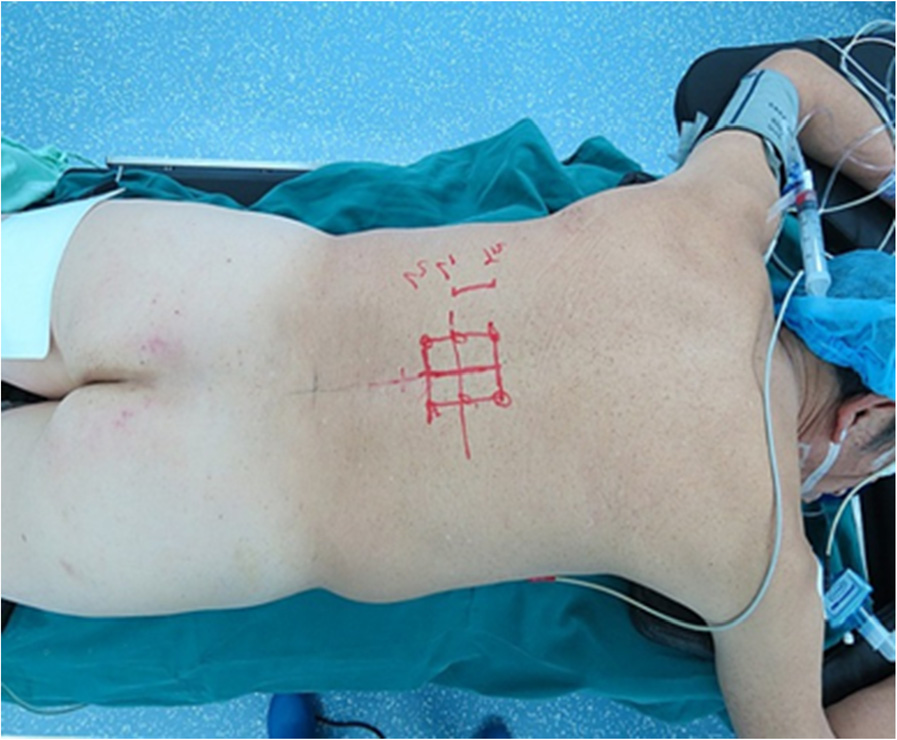
**The marker of incision.**

**Figure 4:**
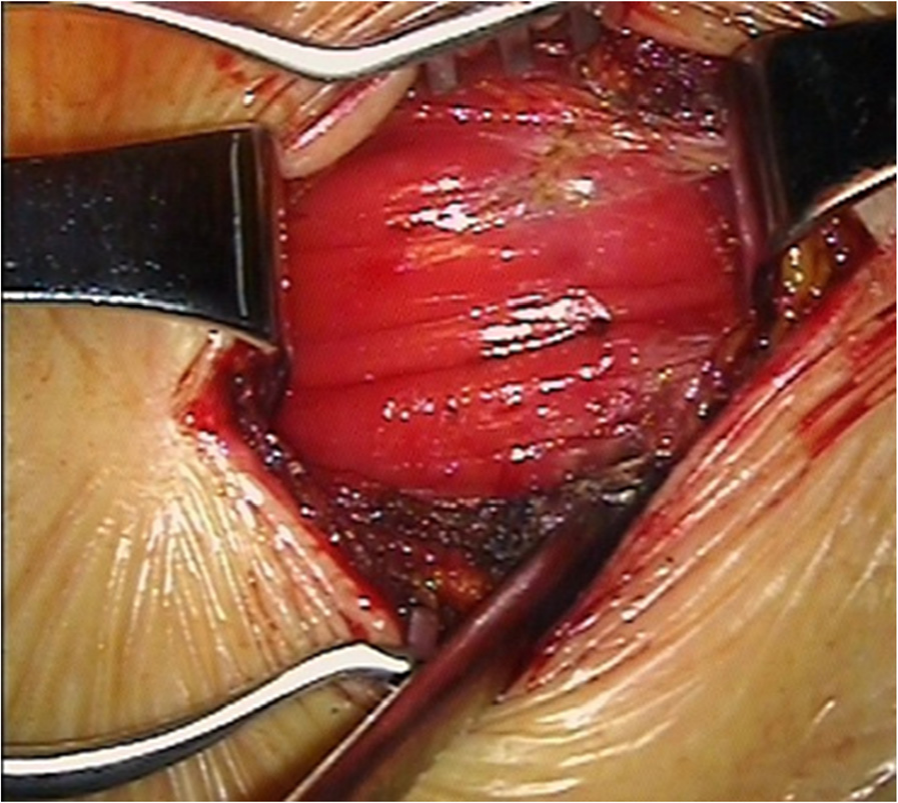
**Exposure of the erector spinae.**

**Figure 5:**
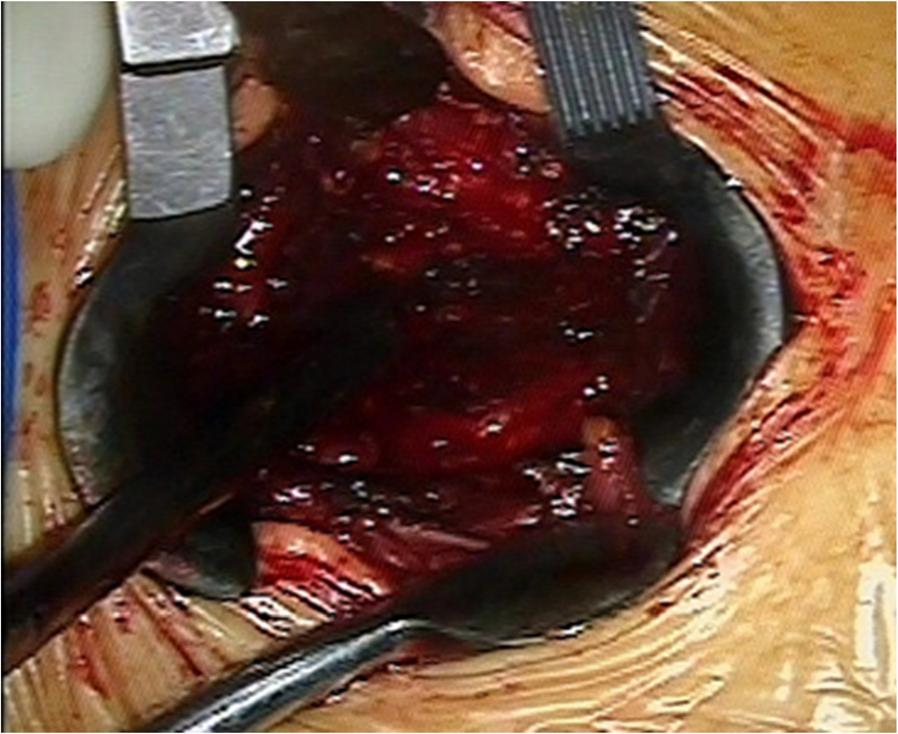
**Minimally invasive surgery retractor.**

The surgical decompression process was performed as follows. After identifying the fractured and adjacent vertebras, longitudinal or transverse incisions were made at the lower level of foramen, 6.5–7.0 cm next to the midline (Figure 3). The minimally invasive surgical retractor was placed after exposure of the lateral iliocostalis (Figures 4 and 5). Decompression was performed at the axillary region of the nerve root (at horizontal angle of approximately 35° to 40°, through the lower part of the intervertebral foramen to reach the injured superior endplate) to remove the bony fragments in the spinal canal. In some cases, the nerve root was retracted modestly and the lower part of the intervertebral foramen was modified by partial removal of the anterior part of the superior articular process and the superior part of the inferior vertebral pedicle to facilitate the decompression. To limit possible interference from the 12th rib decompression was achieved by stretching the erector spinae and adjusting the minimally invasive surgical retractor.

**Figure 6:**
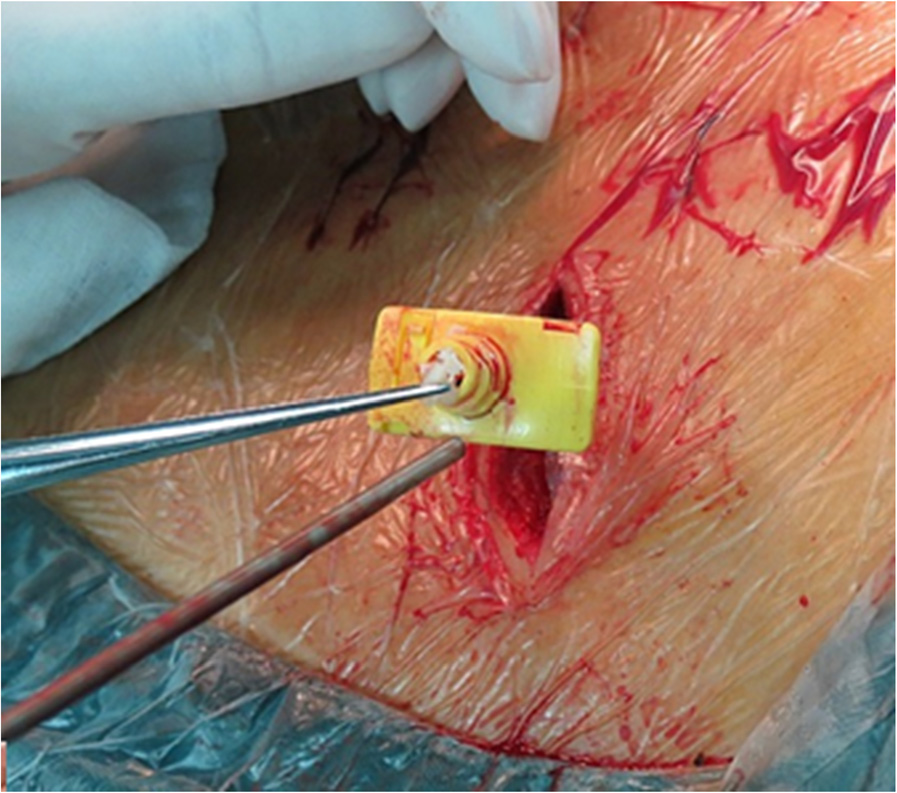
**Implant the artificial bone into injured vertebra.**

**Figure 7:**
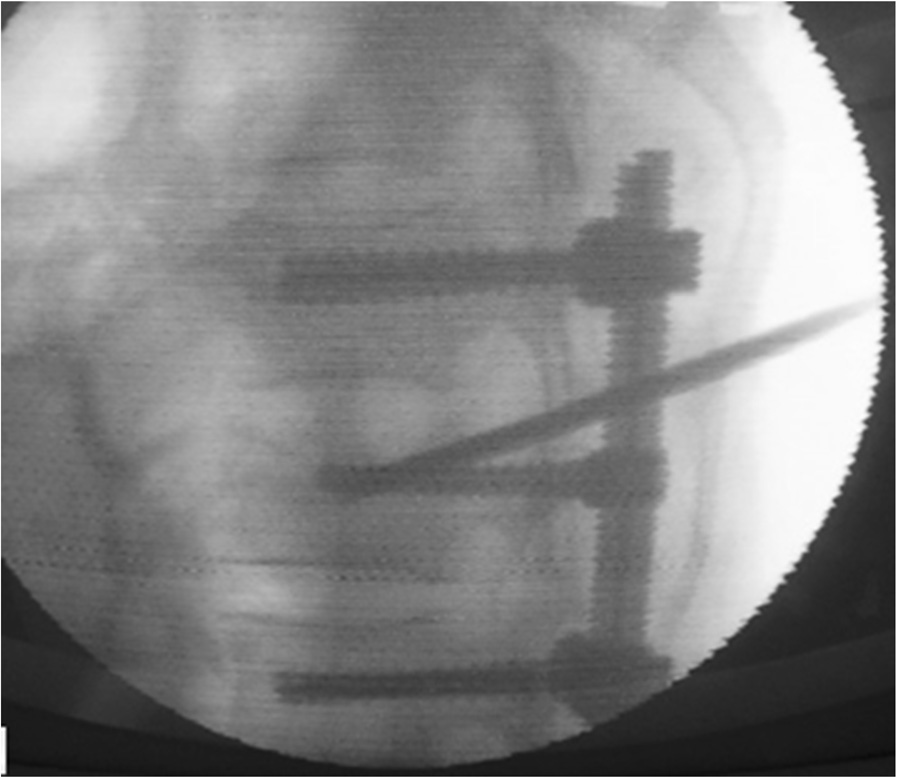
**The lateral perspective before bone graft.**

**Figure 8:**
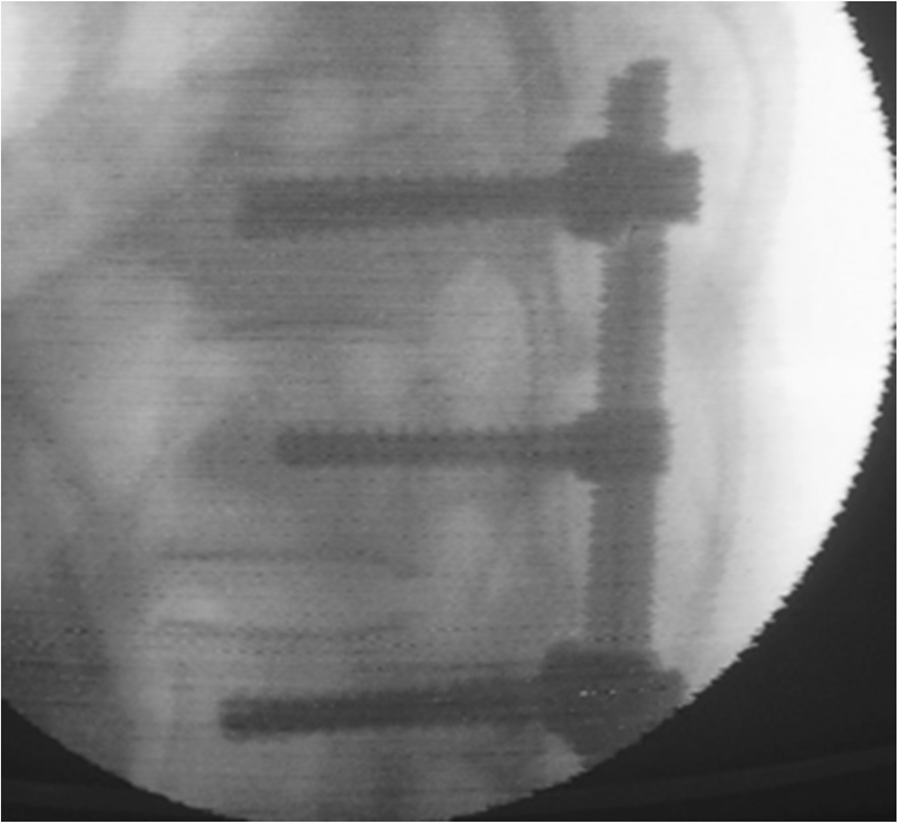
**The lateral perspective after bone graft.**

**Figure 9:**
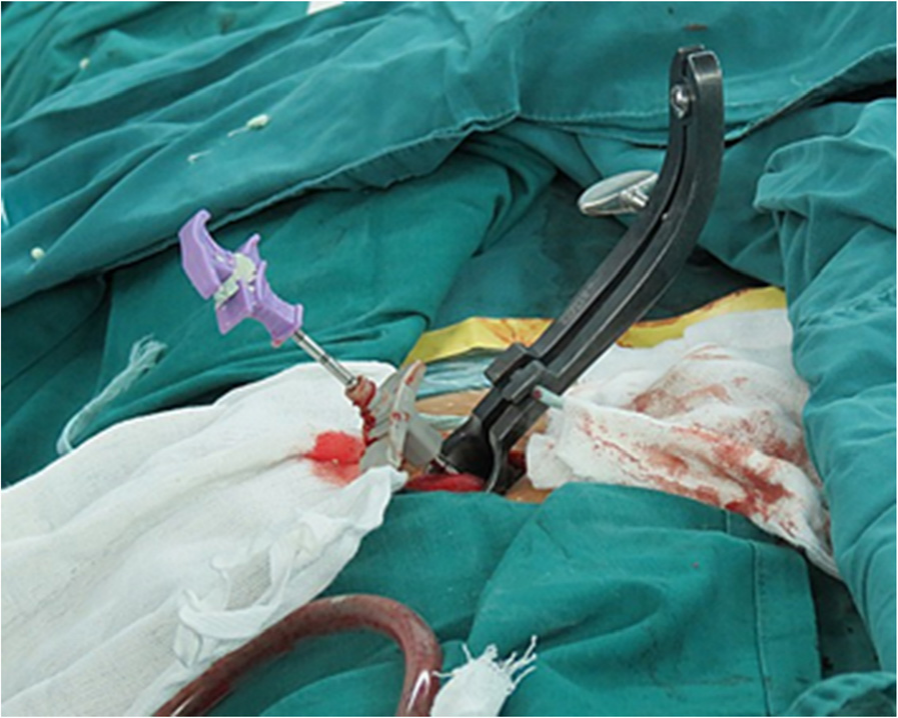
**Injection of the bone cement into the injured vertebra.**

**Figure 10:**
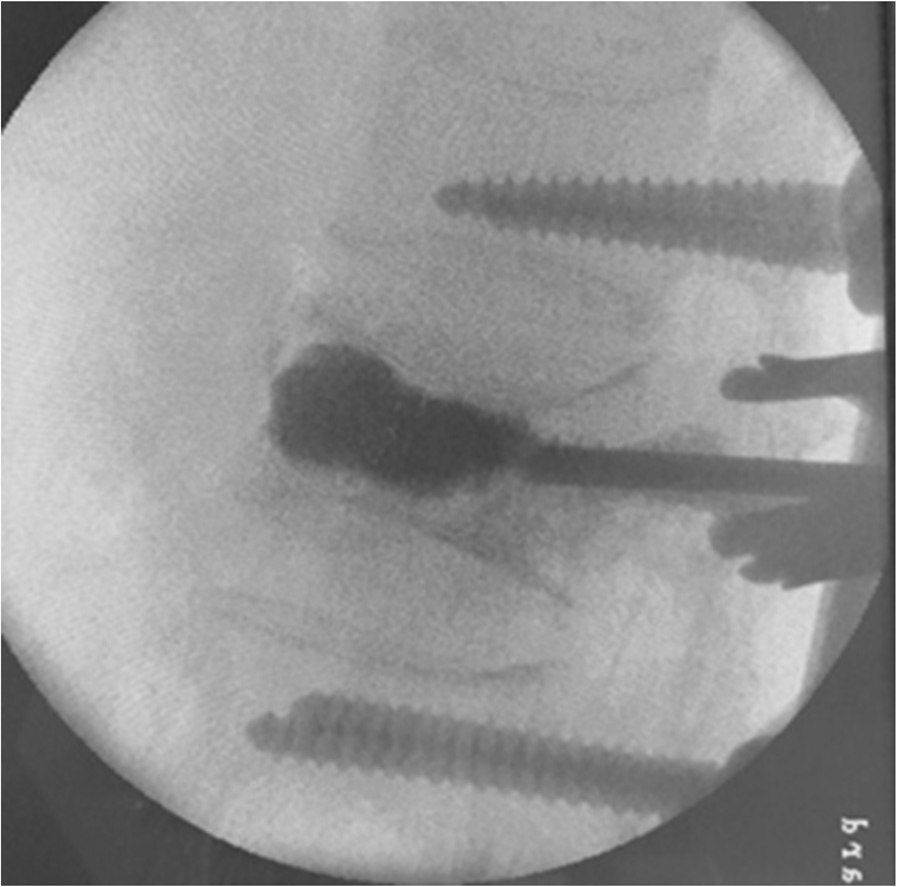
**The lateral perspective.**

**Figure 11:**
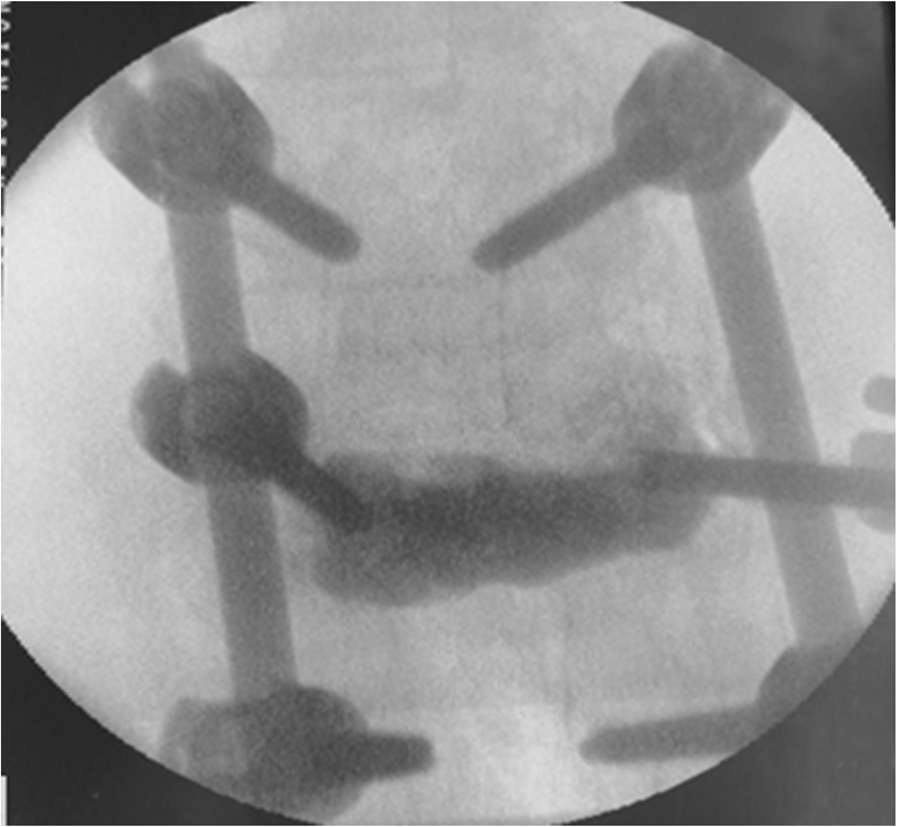
**The anteroposterior perspective.**

Through a paraspinal erector lateral incision, transpedicular vertebral bone graft and bone cement augmentation were completed using percutaneous vertebroplasty (PVP). Among patients with empty vertebral body (*n* = 9), granular bone implant was injected into the vertebral body through a special PVP channel from the lateral top of the pedicle (Figures 6, 7, and 8). For patients presenting with vertebral osteoporosis, without severe damage in the posterior wall (*n* = 5), the same technique was used to unilateral inject bone cement (PMMA) to strengthen the vertebral body. In some cases, since the posterior wall was already impaired, the bone cement injection should be carefully monitored in terms of dosage and timing (Figures 9, 10, and 11).

### Postoperative management

Postoperative use of 20% mannitol was required for 3 days to prevent nerve root edema. The closed drainage was removed 2 days after the operation. The exercise of straight leg raise was recommended 2 days after the surgery. Ambulatory activities while wearing a chest brace were encouraged within 4–6 weeks postoperatively. Patients who underwent bone cement augmentation were allowed to have ambulatory activities within 2–4 weeks after surgery.

### Study outcomes

The duration of surgery and intraoperative blood loss was recorded. All patients were evaluated prior to and immediately after surgery, and at 6 and 12 months postoperatively. X-ray and CT were reviewed for changes in the kyphotic angle, the height ratio of the anterior edge of the injured vertebra (height ratio = height of the anterior edge of the injured vertebra/average height of anterior edge of adjacent vertebras*100%), and the ratio of the sagittal canal compromise (the sagittal canal compromise = loss of the longest sagittal diameter/longest sagittal diameter*100%). Back pain was quantified using a visual analog scale (VAS). Functional outcomes were assessed using the Oswestry Disability Index (ODI) questionnaire and Frankel scores. The VAS and ODI data were recorded prior to and immediately after surgery, and at 6 and 12-month follow-ups. The Frankel scores were collected prior to surgery and at 12 months after surgery.

### Statistical analyses

Statistical analyses were performed using SPSS® statistical package, version 18.0 (SPSS Inc., Chicago, IL, USA) for Windows®. Continuous variables were recorded as the mean ± SD. Between-group comparisons were made using the two-sided Student’s *t*-test for continuous variables. The categorical data were analyzed using the Wilcoxon test. A *p* value <0.05 was considered statistically significant.

## Results

All 21 patients successfully completed the surgery without significant damage to the dura mater, major blood vessels, or other organs. Intraoperative positioning accuracy was achieved, and no accidents such as insertion of screws into the spinal canal occurred during the surgery. No leakage of cerebrospinal fluid or further damage to the nerve root or spinal cord was observed after the surgery. During the 12-month follow-up period, no wound infections, pseudarthrosis, fracture fixation failures, or spinal deformities were reported. The average operation time was 173 min (SD ±23 min). The time for intraoperative vertebral bone cement augmentation (*n* = 7) or vertebral bone graft implantation (*n* = 8) was excluded. The average intraoperative blood loss was 301 ml (SD ±104 ml).

Significant improvement in the kyphotic angle, the height ratio of the anterior edge of the injured vertebra, and the ratio of sagittal canal compromise was noted after the surgery and was maintained during the 12-month follow-up (Table 1).

**Table 1:** **X-radiography and CT data**

**Time**	**Kyphotic angle (°)**	**Height ratio of anterior edge of the injured vertebra (%)**	**Ratio of sagittal canal compromise (%)**
Pre	26.2 ± 8.7	60.0 ± 15.9	46.5 ± 11.4
Post	7.7 ± 4.9*	86.1 ± 8.8*	6.3 ± 4.1*
6-month post	8.8 ± 4.8*	84.9 ± 8.3*	5.3 ± 4.2*
12-month post	9.1 ± 4.7*	84.2 ± 8.6*	4.3 ± 3.6*

Mean ± SD.

*p <0.05 compared with prior to surgery.

**Figure 12:**
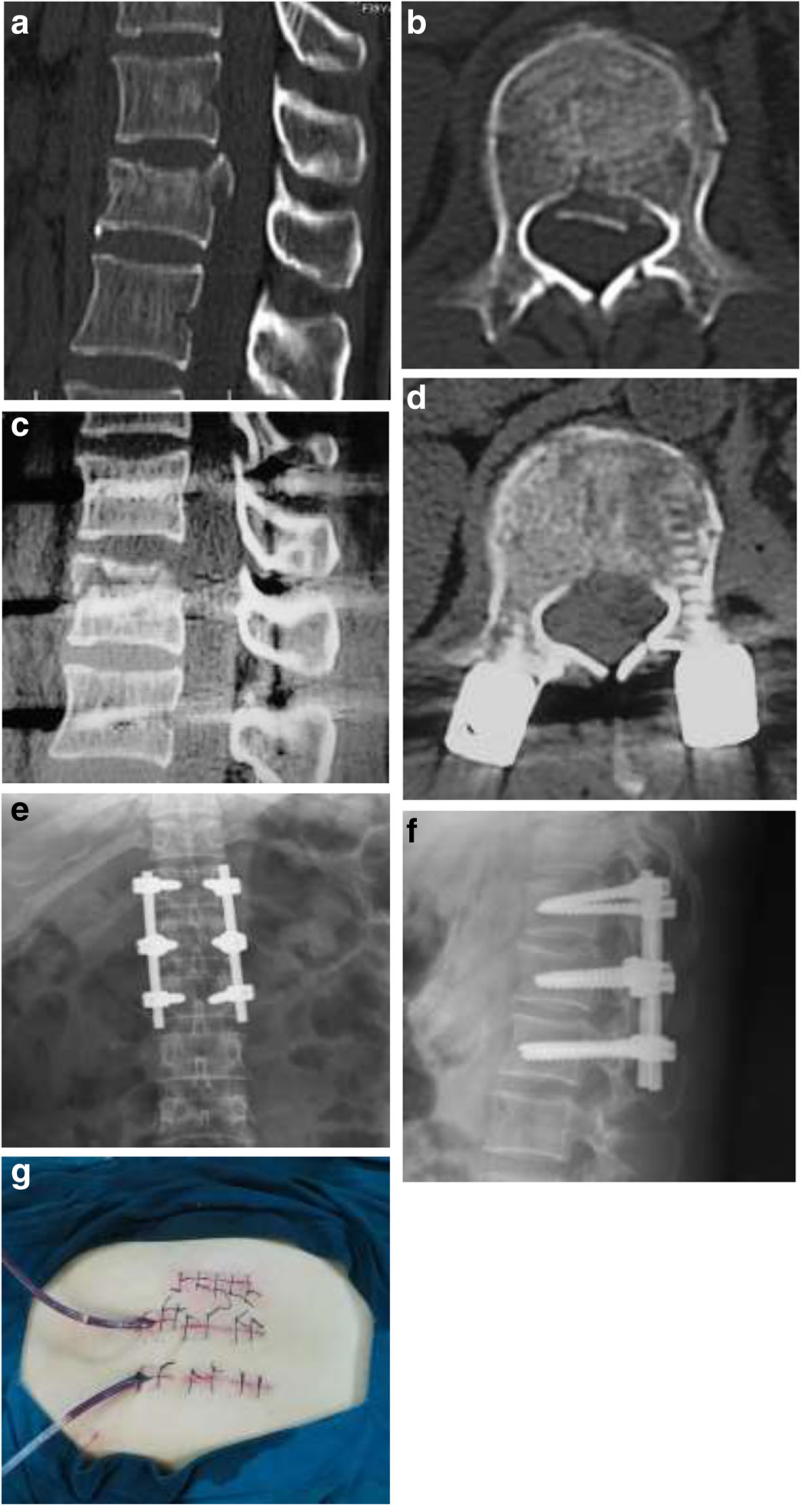
**Images obtained in a 38-year-old woman who underwent direct decompression followed by percutaneous short-segment fixation: (a) Preoperative sagittal sectional CT; (b) Preoperative cross-sectional CT; (c) Postoperative sagittal-sectional CT; (d) Postoperative cross-sectional CT; (e) Postoperative anteroposterior X-ray; (f) Postoperative lateral X-ray; (g) Longitudinal incision next to erector spinae.**

**Figure 13:**
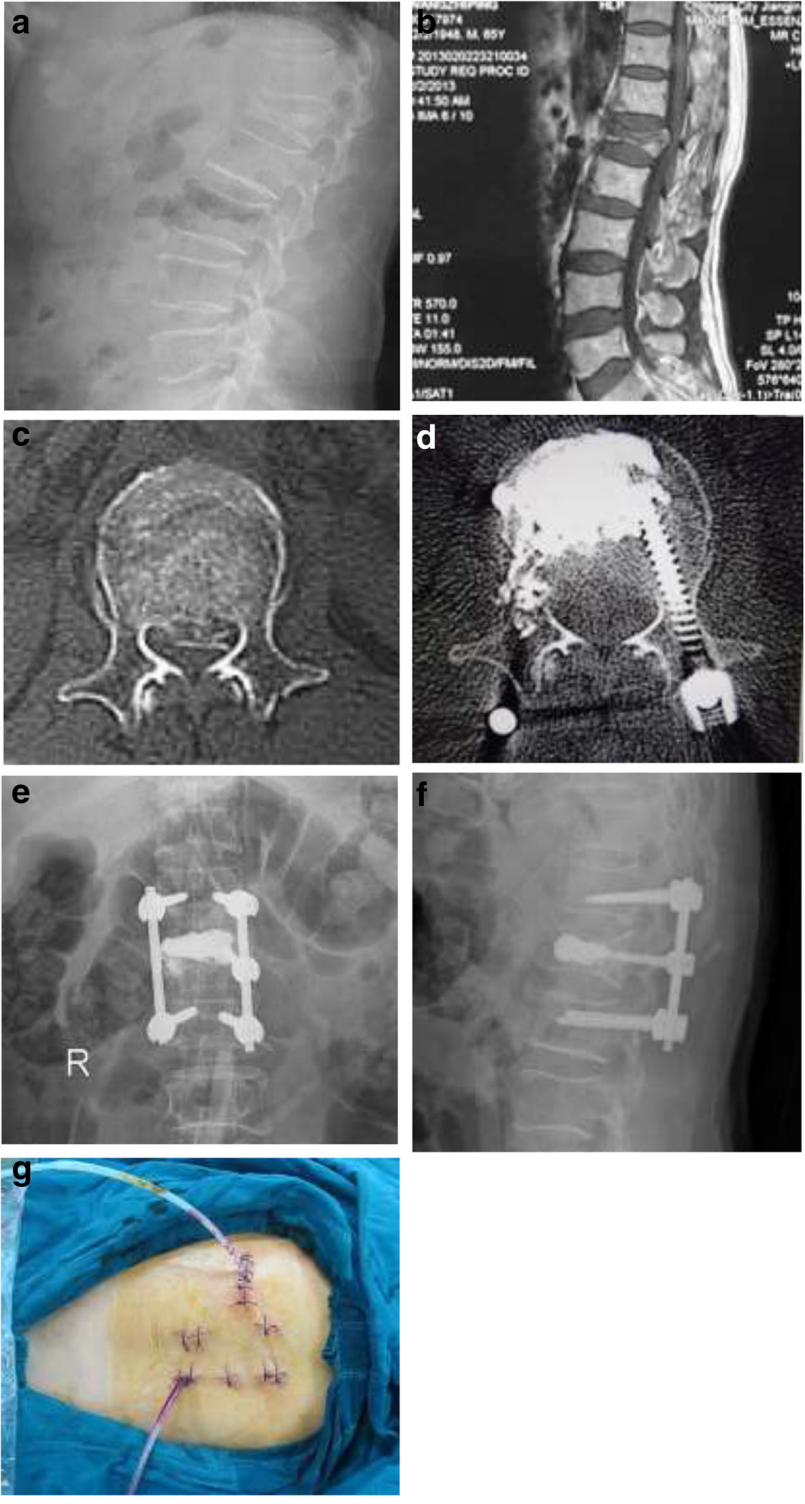
**Images obtained in a 69-year-old man who underwent direct decompression followed by percutaneous short-segment fixation and vertebral augmentation: (a) Preoperative lateral X-ray; (b) Preoperative sagittal-sectional MRI; (c) Preoperative cross-sectional CT; (d) Postoperative cross-sectional CT; (e) Postoperative anteroposterior X-ray; (f) Postoperative lateral X-ray; (g) Transverse incision next to erector spinae.**

Patients reported significantly lower pain levels at 6 months after surgery (VAS score, 7.3 ± 1.2 prior to the surgery versus 2.3 ± 0.9 at 6-month post-surgery; *p* <0.05). VAS scores decreased over time and reached 1.9 ± 0.7 at the 12-month follow-up, significantly lower than the baseline (Table 2). Functional recovery (ODI score) was significantly better at 6 and 12 months after the surgery (baseline: 86.7 ± 5.8 and 28.3 ± 7.6 at 6-month and 16.7 ± 5.1 at 12-month, *p* <0.05). Significant improvement in the functional outcome was also noted using the Frankel score (Table 3, *p* <0.05). Two typical cases are summarized in Table 4 with radiographic assessments presented in Figures 12 and 13.

**Table 2:** **Postoperative back pain quantified using a visual analog scale (VAS) and functional outcome (Oswestry Disability Index (ODI)) in patients with single-level burst fractures of the thoracolumbar spine**

**Time**	**VAS**	**ODI**
Pre	7.3 ± 1.2	86.7 ± 5.8
6-month post	2.3 ± 0.9*	28.3 ± 7.6*
12-month post	1.9 ± 0.7*	16.7 ± 5.1*

Mean ± SD.

*p <0.05 compared with prior to surgery.

**Table 3:** **Improvement in the Frankel score**

**Group**	**Total**	**Pre**					**12-month post**				
		**A**	**B**	**C**	**D**	**E**	**A**	**B**	**C**	**D**	**E**
*n*	21	0	3	5	13	0	0	1	3	5	12

**Table 4:** **Summary of typical cases**

**Case**	**Gender**	**Age**	**Diagnosis**	**Surgery**	**12-month Frankel**	**Figures**
1	Female	38	L1 burst fractures Frankel D	Direct decompression, percutaneous short-segment fixation	E	12–
2	Male	69	L1 burst fractures Frankel B	Direct decompression, percutaneous short-segment fixation, vertebral augmentation	C	13–

Case 1 (Figure 12). Case 2 (Figure 13).

## Discussion

The vertebral burst fractures of Denis B type may commonly involve the vertebral superior endplate, and the bony fragments broken free from the posterior part of the vertebral body may enter the spinal canal, causing symptoms of neurologic deficit [9]. The conventional surgical approach is to make a posterior midline incision to expose the spinous process, followed by stripping paraspinal muscles and stretching the bilateral erector spinae and multifidus muscles to expose the lamina and facet for fixation and decompression [10]. Such an approach has been commonly associated with the spinal nerve damage of the dorsal medial branch and the segmental artery injury of the descending muscular branch, resulting in denervated ischemic atrophy of paraspinal muscle, clinically manifested as flat-back deformity and refractory low-back pain [11]. Numerous studies have demonstrated that spinal muscular atrophy and loss of function are directly associated with failed back surgery syndrome (FBSS) which occurs in 20%–40% of patients undertaking posterior lumbar surgery [12,13].

The erector spinae is a muscle group of the iliocostalis, longissimus, and spinalis. Together with deep multifidus muscle and other muscles, it makes vertebra work more effectively and stabilizes the spine. The conventional posterior surgery approach may potentially cause damage to these muscles, and therefore, protection of these muscles has been proposed for lumbar surgery through a posterior approach [14].

Minimally invasive spine surgery has an increasing emphasis on protection of muscles and posterior ligamentous complex (PLC) to avoid unnecessary injury [15,16]. The percutaneous screw fixation technique was developed as a part of minimal invasive surgery [17,18]. Among patients with spinal burst fractures that bony fragments block the spinal canal, the implementation of this minimally invasive technique largely relies on an indirect decompression after internal fixation with a distraction and disc/ligaments stretch reduction, and decompression is achieved by restoring physiological curve of the spine. The efficiency of this indirect approach is rather limited because the rear structure is not exposed for further decompression. Therefore, we designed the present study to evaluate the paraspinal erector approach in combination with percutaneous screw fixation to achieve more thorough decompression among patients with occupying spinal canal due to burst fractures as a part of minimally invasive surgery.

The paraspinal erector approach in the present study was different from the previously reported dorsal lateral approach, the Wiltse approach, which also involves the erector spinae [19,20]. Although through natural muscle gaps, the Wiltse approach tends to be more inward, with blunt insertions between deep multifidus and longissimus. This approach directly reaches the intervertebral space, suitable for disc repairmen and transforaminal lumbar interbody fusion (TLIF) [21-24]. The Wiltse approach requires a resection of the facet joint to reach the spinal canal, which as an important structural component of PLC bears 10% to 20% of the spinal pressure load [25]. Therefore, the resection of the facet joint will significantly reduce the stability of the lumbar motion segment, which appears to be important for patients with burst fractures without anterior fusion. The paraspinal erector approach can avoid this pitfall. In addition, to reach the front of the spinal canal, the Wiltse approach has to stretch the dural sac and nerve root, which significantly increases the risk of surgical injury above L2 segments.

The paraspinal erector approach theoretically has the following advantages: (1) insertion from the lateral edge of the erector spinae with less muscle stripping and injury; (2) through the intervertebral foramen, directly reaches the spinal canal, having less structural damage on the facet and lamina, keeping the PLC integrity; (3) through the inferior part of intervertebral foramen directly reaching the front of the canal in a relatively large angle of inclination, therefore, adequately decompress the central, lateral, and foraminal zones of the spinal canal with little impact on the neural elements. Furthermore, the nerve root in the extraforaminal areas has a larger movable degree than that in the foramina. Thus, compared with the conventional posterior approach, the possibility of surgical neural injury caused by the stretching of the nerve root would be remarkably reduced.

In the present study, we chose patients with L1 and L2 fractures as the target population for this new approach for several reasons. Burst fractures commonly occur in thoracolumbar (T11-L2). In the thoracic segments, since the endpoint of iliocostalis is attached to the costal angle, the lateral approach is generally blocked by the lower part of the ribs. Our previous anatomical studies indicated that a different approach was needed, with the starting point adjustment from the lateral edge of the iliocostalis to the longissimus that could reach the spinal canal through intervertebral foramen by stretching the longissimus to the medial [8]. While our present study focused on upper lumber burst fractures, due to the remarkable differences between lumbar and thoracic vertebrae, further research of this approach in thoracic fractures is warranted.

In this study, we completed percutaneous pedicle screw posterior fixation and spinal canal direct anterior decompression in 21 patients with Denis B type upper lumbar burst fractures. This surgical approach effectively corrected the spinal kyphotic angle, restored vertebral height, and significantly reduced the canal sagittal diameter compromise. The mean blood loss of 300 ml was significantly lower than that of anterior corpectomy and Z-plate fixation (approximately 1,700 ml) [26] but similar to that of short-segment pedicle instrumentation (approximately 430 ml) [27]. Postoperative VAS and ODI values decreased significantly, indicating reduced surgical pain and less functional impairment, which suggest the advantage of this approach with limited injuries in the paraspinal muscles and posterior column. The improvements of pain and function associated with this approach compared favorably with previous outcomes of posterior approach [28]. The changes in the Frankel classification also confirmed the significant effect of this approach on neurological function recovery. The findings demonstrate the feasibility and clinical utility of direct spinal canal decompression as a part of minimally invasive surgery. Additionally, the paraspinal erector approach would allow further bone graft and bone cement augmentation, utilizing PVP technology through the lateral top of the pedicle into the vertebral body. Combined with the percutaneous pedicle screw fixation, this approach can also be used for discectomy and interbody fusion (data not shown).

In conclusion, this new approach was successfully used for patients with upper lumber burst fractures resulting favorable outcomes in improvements of pain and function, kyphotic deformity correction, reasonable surgical time, and minimal blood loss. However, it requires in-depth anatomy and surgical skills and accordingly longer surgical training. This study also has some limitations such as small sample size and relatively short follow-up. Therefore, the clinical utility of this new surgical approach warrants a confirmation in clinical trials of patients with spinal fractures.
